# Mitochondrial Proton Leak Compensates for Reduced Oxidative Power during Frequent Hypothermic Events in a Protoendothermic Mammal, *Echinops telfairi*

**DOI:** 10.3389/fphys.2017.00909

**Published:** 2017-11-10

**Authors:** Elias T. Polymeropoulos, R. Oelkrug, M. Jastroch

**Affiliations:** ^1^Institute for Marine and Antarctic Studies, University of Tasmania, Hobart, TAS, Australia; ^2^Center of Brain, Behavior and Metabolism, University of Lübeck, Lübeck, Germany; ^3^Institute for Diabetes and Obesity, Helmholtz Zentrum München, Munich, Germany; ^4^Helmholtz Diabetes Center, German Center for Diabetes Research (DZD), Neuherberg, Germany

**Keywords:** mitochondria, proton leak, lesser hedgehog tenrec, endothermy, protoendotherm, cold acclimatization

## Abstract

The lesser hedgehog tenrec (*Echinops telfairi*) displays reptile-like thermoregulatory behavior with markedly high variability in body temperature and metabolic rate. To understand how energy metabolism copes with this flexibility, we studied the bioenergetics of isolated liver mitochondria from cold (20°C) and warm (27°C) acclimated tenrecs. Different acclimation temperatures had no impact on mitochondrial respiration using succinate as the substrate. Mimicking the variation of body temperature by changing assay temperatures from 22 to 32°C highlighted temperature-sensitivity of respiration. The 40% reduction of respiratory control ratio (RCR) at 22°C compared to 32°C, a common estimate for mitochondrial efficiency, was caused by reduced substrate oxidation capacity. The simultaneous measurement of mitochondrial membrane potential enabled the precise assessment of efficiency with corrected respiration rates. Using this method, we show that proton leak respiration at the highest common membrane potential was not affected by acclimation temperature but was markedly decreased by assay temperature. Using membrane potential corrected respiration values, we show that the fraction of ATP-linked respiration (coupling efficiency) was maintained (70–85%) at lower temperatures. Collectively, we demonstrate that compromised substrate oxidation was temperature-compensated by the reduction of proton leak, thus maintaining the efficiency of mitochondrial energy conversion. Therefore, membrane potential data suggest that adjustments of mitochondrial proton leak contribute to energy homeostasis during thermoregulatory flexibility of tenrecs.

## Introduction

Endotherms like mammals and birds display the unique ability to maintain a high body temperature (T_b_) over a wide ambient temperature (T_a_) range (Crompton et al., 1978). Unlike in ectotherms, where T_b_ is generally dependent on T_a_, this evolutionary strategy has enabled endotherms to expand into highly specialized niches and a wide geographical distribution. Despite the establishment of the gross classification into endo- and ectothermic animals, the distinction is often not clear cut, with several endothermic taxa displaying distinct ectothermic physiological traits and vice versa.

The lesser hedgehog tenrec (*Echinops telfairi*) as a prime example, displays reptile-like thermoregulatory behavior with markedly high variability in T_b_ and metabolic rate (Scholl, 1974; Lovegrove and Génin, 2008; Oelkrug et al., 2013). The tenrec displays one of the lowest normothermic T_b_ (~32°C) compared to all other placental mammals in winter conditions and exhibits regular daily torpor behavior with substantial reductions in T_b_ (following reductions in T_a_) and metabolic rate. Extraordinarily, *E. telfairi* females only display constantly high T_b_ during reproduction and parental care (Poppitt et al., 1994). Hence, this afrotherian species is referred to as protoendothermic, representing an intermediate state of thermoregulatory rigor compared to more “modern” homeothermic eutherian species. This thermoregulatory setup of protoendotherms is therefore often considered a representative characteristic of the evolutionary transition from ecto- to endothermy (Lovegrove and Génin, 2008; Mckechnie and Mzilikazi, 2011).

Interestingly, the basal metabolic rate (BMR) of the tenrec is similar to ectothermic metabolic rates of the same body mass, while the basal mitochondrial proton leak in the liver, a significant contributor of metabolic heat production (Brand et al., 1994; Rolfe and Brown, 1997), is at an intermediate level between endo- and ectotherms (Polymeropoulos et al., 2017).

Prolonged cold exposure triggers complex physiological adjustments to ensure cellular homeostasis at a constant T_b_ in many mammals. There is evidence that *E. telfairi* is capable of non-shivering thermogenesis mediated by functional brown adipose tissue (BAT) and hence of a mechanism that is unique to “modern” eutherian mammals (Oelkrug et al., 2013, 2015). The bioenergetic setup of tenrec mitochondria in other tissues may also be highly advanced and equipped to adjust energy metabolism in response to environmental stressors such as low ambient temperatures.

While UCP1-mediated non-shivering thermogenesis in BAT is the most prominent mechanism to enable survival in the cold and arousal from torpid or hibernating states of small eutherian mammals (Cannon and Nedergaard, 2004; Oelkrug et al., 2011), mitochondrial adjustments in other tissues of high oxygen uptake have also been described. It appears that another important thermogenic tissue is skeletal muscle which contributes with shivering and possibly other alternative mechanisms of non-shivering thermogenesis using SERCA pathways (Rowland et al., 2015). The liver is commonly studied to assess the relationship between metabolism and mitochondrial bioenergetics as the liver contributes significantly (10–20%) to the metabolic rate of mammals (Field et al., 1939) and mitochondria are easy to isolate as compared to e.g., skeletal muscle.

In general, mitochondria convert nutritional energy into cellular energy by oxidative phosphorylation, a process that consumes about 90% of the cellular oxygen uptake in mammals (Rolfe and Brown, 1997). Mitochondrial substrates are oxidized and their electrons are transported along the respiratory complexes before reducing oxygen to water. The potential energy of the electrons is used to pump protons out of the matrix, generating a proton motive force that drives the ATP synthesis. The energy transduction to ATP is, however, not fully efficient as the proton motive force is also consumed by the mitochondrial proton leak (Jastroch et al., 2010). The management of the energy budget of an animal can be adjusted by alterations in mitochondrial efficiency when exposed to environmental stress, either by a decrease of substrate oxidation or by changes in proton permeability as shown for many ectotherms (Gnaiger et al., 2000; St-Pierre et al., 2000; Trzcionka et al., 2008).

During episodes of energy shortage such as cold seasons or food scarcity, small mammals use energy conserving mechanisms such as torpor or hibernation to decrease energy demand by dramatic reduction of T_b_ and metabolic rate. During torpor and hibernation, state 3 (phosphorylating) respiration and maximal substrate oxidation are reversibly suppressed in isolated liver mitochondria (Staples and Brown, 2008; Kutschke et al., 2013). The degree of suppression is, not surprisingly, highly dependent on experimental assay temperature. Interestingly, it appears that the suppression of state 3 respiration in hypothermic states is only detectable above a critical assay temperature of 30°C. Above 30°C assay temperature, passive thermal effects rather than active suppression may become more important for metabolic rate (Staples and Brown, 2008). This is further supported by the lack of active suppression in isolated liver mitochondria of the Golden spiny mouse (*Acomys russatus*), a desert species that displays significant reduction of energy metabolism without pronounced reduction of T_b_ (Grimpo et al., 2014). The findings at lower assay temperatures may well be related to changes of the biophysical properties of mitochondrial membranes.

In isolated liver mitochondria of the homeothermic rat (*Rattus norvegicus*), lowering the assay temperature from 37 to 25°C reduces state 3 (phosphorylating) and state 4 (non-phosphorylating) respiration by 50%, (Dufour et al., 1996). In the heterothermic Djungarian hamster (*Phodopus sungorus*), substrate oxidation of isolated liver mitochondria is suppressed in torpid animals (T_b_ < 31°C). The suppression of substrate oxidation affects respiration rates the most at normothermic assay temperatures (37°C) but not at torpor-like assay temperatures (15°C). At 15°C, mitochondrial proton leak is increased in mitochondria of torpid but not normothermic animals (Brown et al., 2007). The respiration of liver mitochondria correlates with body temperature but there is no suppression found for kidney, skeletal muscle, and heart mitochondria, suggesting tissue-specificity of mitochondrial suppression (Kutschke et al., 2013).

Given these findings in rodents, it may be expected that frequent body temperature changes of the tenrec affect mitochondrial respiration through passive Q_10_ driven changes but it is unknown whether mechanistic alterations in response to cold exposure, such as active suppression, contribute as well.

While the general thermoregulatory patterns of the lesser hedgehog tenrec, a representative protoendothermic species, have become more transparent through recent research, underlying function or mechanisms that have co-evolved with thermoregulatory flexibility, remain unclear. Here, we studied the bioenergetics of isolated liver mitochondria of cold vs. warm acclimated, non-torpid tenrecs at different assay temperatures to understand functional adjustments that may assist the animals to manage energy homeostasis during the transition to various body temperatures.

## Materials and methods

### Animal husbandry

Laboratory bred male (*n* = 4) and female (*n* = 8) lesser hedgehog tenrecs (*E. telfairi*) were measured during their annual activity period. Throughout the experiment, animals were housed individually in Typ IV makrolon cages (1,800 cm^2^) with sawdust bedding and plastic nest boxes and were kept on a 12:12 h light-dark cycle at an ambient temperature of 23 ± 1°C. They had free access to water and were fed with canned cat food (KiteKat), cockroaches, seeds and fruits every second day at 2–5 p.m. All experimental procedures were approved by the German Animal Welfare Authorities (Regierungspräsidium Gießen, Hessen, MR 17/1-Nr.116/2010).

### Body temperature recordings

For continuous body temperature recordings on freely moving animals, implantable radio transmitters and receiver plates (Model X; Mini Mitter, Sunriver, OR, USA; accuracy 0.1°C) were used as described in detail previously (Oelkrug et al., 2013). After implantation animals were allowed to recover for at least 3 weeks before one group of animals was sequentially acclimated to 20°C (4 days at 24°C, 3 days at 21–22°C; cold acclimated group = CA group; *n* = 6) while the other group remained at 27°C (warm acclimated group = WA group; *n* = 6) (Oelkrug et al., 2013). After an acclimation period of 2 weeks, core body temperature (±1°C) was recorded every 6 min for 7–12 days at 27°C and 3–6 days at 20°C (only CA group).

### Minimum and resting metabolic rate

To assess the impact of ambient temperature on energy turnover in the tenrecs, the minimum (MR_min_) and resting metabolic rate (RMR) were determined over a temperature range of 14°C (20–34°C) in tenrecs acclimatized to 20°C (CA) or 27°C (WA) using a two-channel respiratory system as described previously (Oelkrug et al., 2013). Briefly, tenrecs were exposed for 4–5 h per day to one randomly selected ambient temperature and readings of MR (O_2_ consumption) and T_b_ were taken every 3–5 min. At ambient temperatures below 30°C animals immediately decreased their metabolism to minimum values (MR_min_), resembling a torpor-like state, and had to be disturbed by the introduction of food to their cage (RMR, non-post absorptive) to obtain resting, non-torpid metabolic rate.

### Mitochondrial isolation

Before sacrifice animals were disturbed and allowed to reach normothermic T_b_ (>30°C). The rewarming took less than 30 min and after additional 30 min at normothermia the animals were sacrificed and their livers were harvested. Liver mitochondria were isolated by homogenization in STE buffer (250 mM sucrose, 5 mM Tris, 2 mM EGTA, pH 7.4 at 4°C) using a glass-glass-homogenizer, followed by differential centrifugation (see Trzcionka et al., 2008 for further details). All steps of mitochondrial isolation were performed on ice or at 4°C. After isolation, mitochondrial protein content was determined photometrically using the Biuret method (Gornall et al., 1949) and fatty acid free bovine serum albumin as standard. Measurements were started 45 min after mitochondria preparation.

### Mitochondrial respiration rates

Mitochondrial oxygen consumption was measured with a Clark-type electrode connected to a temperature controlled water bath and calibrated with air-saturated medium [KHE buffer: 120 mM KCl, 5 mM KH_2_PO_4_, 3 mM HEPES, 1 mM EGTA, 0.3% bovine serum albumin (BSA; w/v), pH 7.2 at RT] which was assumed to contain 432 nmol O/ml at 32°C (Reynafarje et al., 1985). Mitochondria were diluted to 1.5 mg/ml mitochondrial protein in 500 μl KHE buffer and rotenone (4.8 μM) was added to inhibit complex I of the respiratory chain. Afterwards mitochondrial respiration was started by adding 4 mM succinate (state 2), followed by 600 μM ADP (state 3), and 1 μg/ml oligomycin (state 4). At the end of each measurement FCCP (carbonyl cyanide-p-triuoromethoxyphenylhydrazone) was added to induce maximum substrate oxidation.

The respiratory control ratio (RCR), determined by calculating the quotient of state 3 and state 4 respiration, was measured to ascertain the integrity of isolated mitochondria.

Temperature-dependency of mitochondrial respiration rates was calculated by performing measurements at different assay temperatures (22, 27, and 32°C).

### Mitochondrial proton leak kinetics and coupling efficiency

The kinetics of mitochondrial proton leak were measured by sequentially inhibiting respiration that drives the proton leak and plotting respiration rates against their corresponding membrane potentials. Measurements were performed with 1.5 mg/ml of liver mitochondria in KHE buffer. Mitochondrial membrane potential was measured simultaneously to mitochondrial respiration by using a TPMP^+^ (triphenylmethylphosphonium)—sensitive probe. State 4 potentials were assessed in the presence of 100 nM nigericin (Cadenas and Brand, 2000), 4.8 μM rotenone, and 1 μg/ml oligomycin. At the beginning of each measurement, sequential additions of TPMP^+^ up to 2.5 mM served to calibrate the TPMP^+^ -sensitive electrode. Mitochondrial oxidation was initiated afterwards by the addition of 4 mM succinate and progressively inhibited with malonate up to 11.3 mM to establish decreasing steady state membrane potentials. Finally, FCCP was added to dissipate the membrane potential and release TPMP^+^ from the mitochondria, allowing for correction of baseline drift.

State 3 potential was measured using the same experimental setup. First, 4.8 μM rotenone was added to inhibit complex I, followed by 4 mM succinate (state 2), 600 μM ADP (state 3), 50 nM nigericin (state 3), and finally FCCP for correction.

The simultaneous measurement of mitochondrial membrane potential enabled to compare mitochondrial electron flux of state 3 and proton leak at the same steady state potential. Thus, we determined the oxygen consumption that either drives the ATP synthase or drives the mitochondrial proton leak. What proportion of the total electron flux is dedicated to the synthesis of ATP, while the rest is lost as heat through the proton leak, that is defined as coupling efficiency (efficiency of respiration “coupled” to ATP synthesis)(Affourtit and Brand, 2009). In our measurements, this is calculated as ATP-linked respiration (state 3 minus proton leak) divided by total respiration (state 3).

This determination was performed at assay temperatures of 22, 27, and 37°C.

### Statistical analysis

Body mass between WA and CA groups was tested for differences using the *t*-test. The Mann-Whitney test for two independent samples was used to test for differences of T_b_ frequencies between T_a_ = 20°C and T_a_ = 27°C. RMR or MR_min_ values, and corresponding T_b_ values were tested for differences at various T_a_ using a two-way repeated measures analysis of variance (ANOVA) approach, considering acclimation (20 vs. 27°C) and T_a_ as factors. Similar analysis was performed considering T_a_ and metabolic rate (RMR vs. MR_min_) as factors. Changes in RMR, MR_min_, and T_b_ with changes in T_a_ were further analyzed by standard least squares linear regression. The slopes of the linear regressions (m) were tested for significance using ANOVA. Mitochondrial respiration rates and RCR between acclimation groups or between assay temperature changes were tested for differences using two-way repeated measures ANOVA and least squares regression for changes with T_a_ in addition. HCP is defined as Highest Common mitochondrial membrane Potential of all proton leak curves that are compared. The oxygen consumption driving proton leak at HCP is estimated based on the leak kinetics, assuming linearity between two adjacent proton leak measurements of the curve. Coupling efficiency at different assay temperatures was analyzed using least squares linear regression. All pairwise multiple comparisons procedures were performed using adjusted Bonferroni *post-hoc* modified *t*-tests.

## Results

### Body mass, metabolic rate, and T_b_

Liver mitochondria were isolated from *E. telfairi* individuals for which post- experimental body mass, metabolic rate, and T_b_ have been assessed previously (Oelkrug et al., 2013). Briefly, acclimation did not impact body mass which was 159 ± (*SD*) 17 g for 20°C and 132 ± (*SD*) 23 g for 27°C acclimated animals.

**Figure 1:**
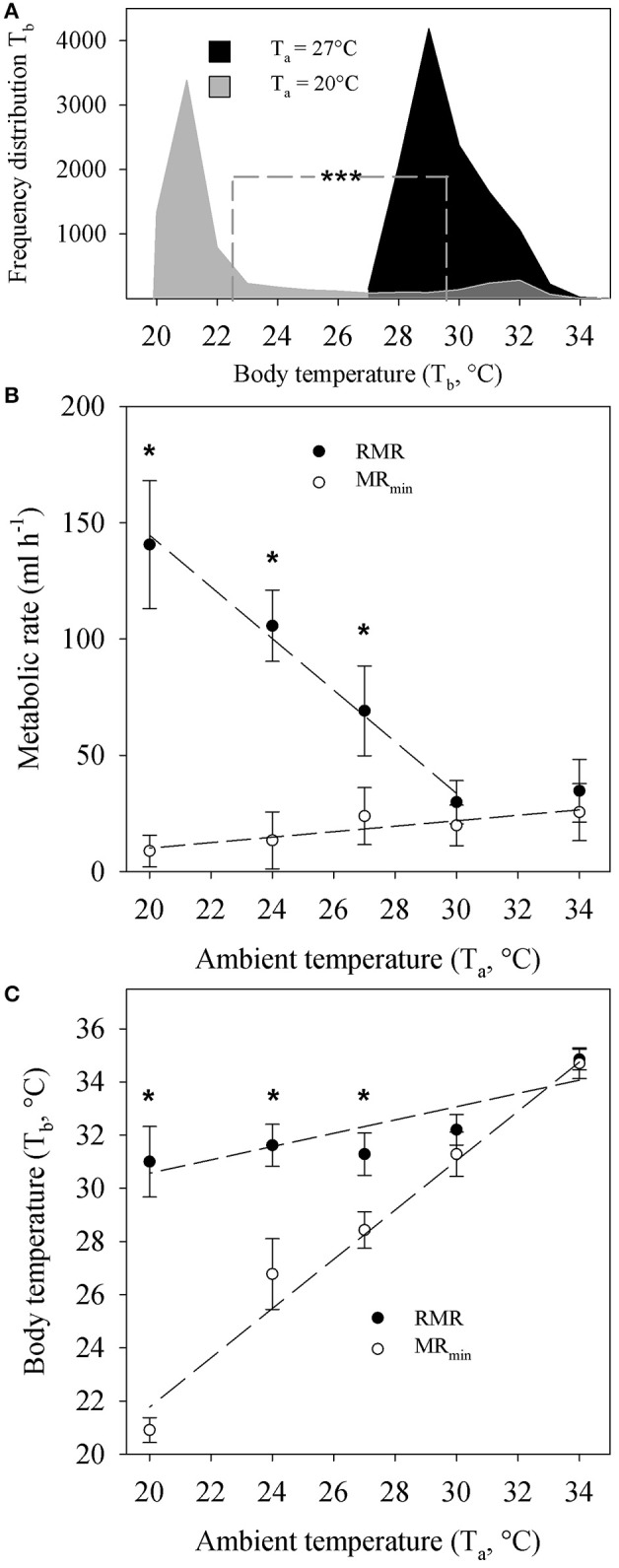
**(A)** Frequency distribution of repeatedly measured core body temperature recordings (T_b_) of *Echinops telfairi* exposed to 20°C (light gray, 3–6 days, *n* = 6) and 27°C (black, 7–12 days, *n* = 6) ambient temperatures (T_a_). Dashed line indicates mean values for each temperature, which were significantly different from each other (Mann-Whitney, ^***^P < 0.001). **(B)** Temperature dependent resting metabolic rate (RMR) and minimum metabolic rate (MR_min_) of *Echinops telfairi* at different T_a._ There was no significant difference in RMR or MR_min_ between animals acclimated to 20 or 27°C. Hence the data presented here is the average ± SE of both groups. **(C)** Temperature dependent T_b_ changes during measurements of RMR and MR_min_ above. Values are means ± SE. ^*^ indicates significant differences between measurements of MR and MRmin and T_b_ at different T_a_ (two-way repeated measures ANOVA, *P* < 0.05). Dashed lines for **(B**,**C)** denote linear regressions fits to the data of the form y = mx + b and the *P* values indicate where the slope of the regression (m) was significantly different from zero (ANOVA, *P* < 0.05). RMR: y = −11.098x + 366.52, m: *P* = 0.005; MR_min_: y = 1.18x−13.4, m: *P* = 0.039; T_b_ at RMR: y = 0.2474x + 25.571, m: *P* = 0.049; T_b_ at MR_min_: y = 0.9277x + 3.204, m: *P* = 0.002. Note, the value for RMR at 34°C was not included in the linear regression analysis for RMR as it did not significantly differ from the value at 30°C. Hence, these two data points are within the TNZ for this species. The content of this figure was adapted from Oelkrug et al. (2013).

For the purpose of this study we re-analyzed core body temperature data (T_b_, °C). T_b_ in tenrecs kept at 20°C was different to T_b_ in animals at 27°C (Figure 1A). The frequency distribution for T_b_ at T_a_ = 20°C peaked at 21°C while the distribution at 27°C peaked at 29°C, which also equals the median values at each T_a_. The mean value for T_b_ at T_a_ = 20°C was 22.5 ± 3.4 and 29.6 ± 1.3°C at T_a_ = 27°C and was significantly different.

RMR data from Oelkrug et al. (2013) revealed significant increase of metabolism at T_a_ below 30°C (Two-way ANOVA, *P* ≤ 0.01, Figure 1B). The RMR between 30 and 34°C was similar, indicating that both values are within the thermoneutral zone (TNZ) of the tenrec. Here, we re-analyzed the data by applying a linear regression analysis and confirmed a significantly negative correlation coefficient for RMR with increasing T_a_ (Figure 1B). MR_min_ showed a small but significant positive regression coefficient with increasing T_a_ from 20 to 34°C but MR_min_ values were significantly lower (Two-way ANOVA, *P* ≤ 0.01) compared to RMR below a T_a_ of 30°C, and below the TNZ. There were no significant differences between acclimation groups at each T_a_ for RMR or MR_min_, hence the data were pooled for the analyses and figures.

T_b_ during measurements of RMR significantly decreased with decreasing T_a_ by overall 2.8°C (Figure 1C). During measurements of MR_min_ on the contrary, the absolute decrease in T_b_ was 13.8°C. Here, T_b_ tracked T_a_ closely as T_a_ decreased with a scaling coefficient of 0.93 that was statistically, significantly different from zero (*P* = 0.039). There also was a statistically significant difference in T_b_ between RMR and MR_min_ measurements below a T_a_ of 30°C, corresponding to changes in MR_min_ (Figure 1C).

### Mitochondrial respiration

**Figure 2:**
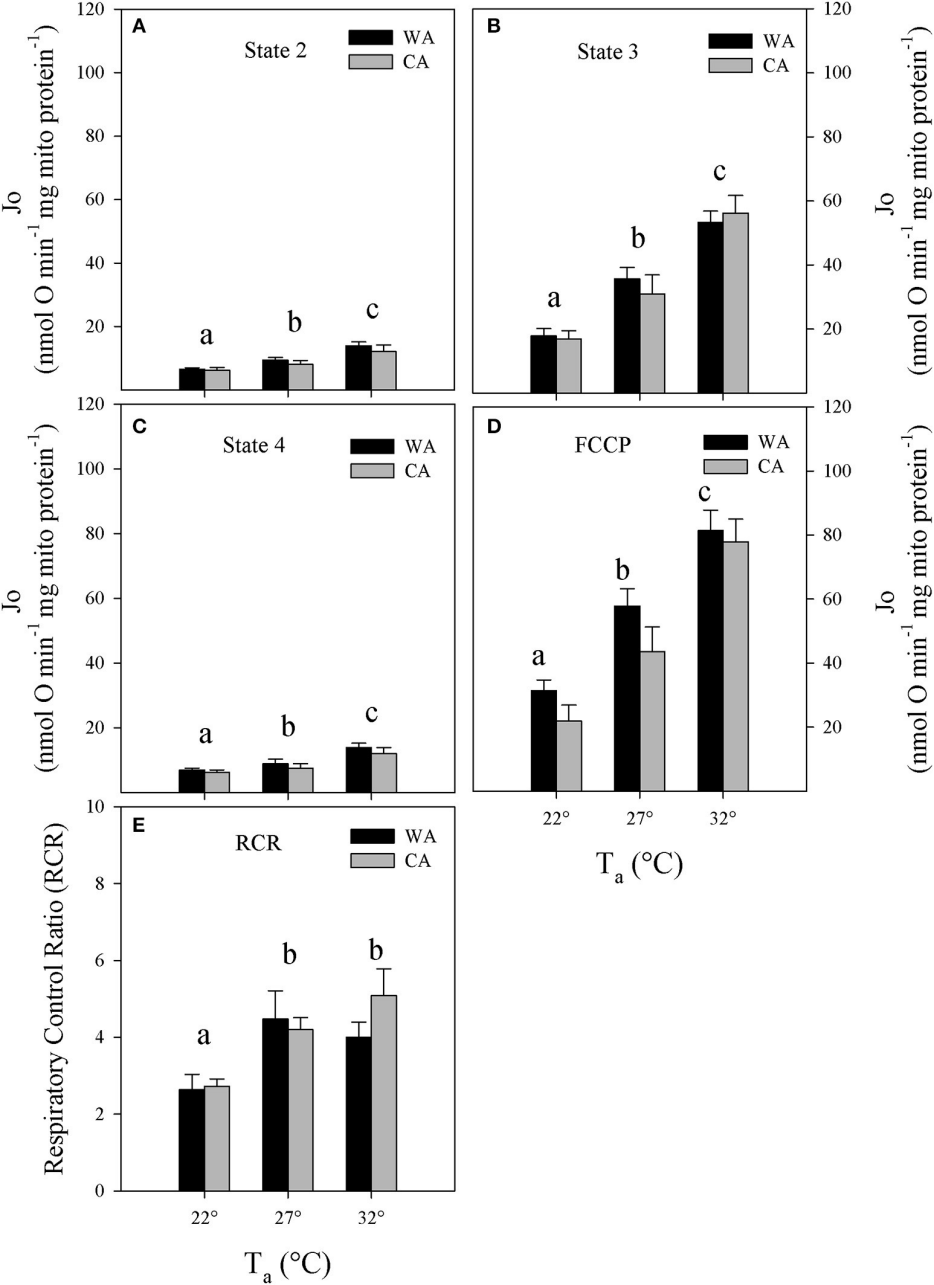
Mitochondrial respiration rates (Jo) at **(A)** state 2, **(B)** 3, and **(C)** state 4; **(D)** upon chemical uncoupling with FCCP as well as **(E)** corresponding respiratory control ratios (RCR) of liver mitochondria, isolated from *Echinops telfairi* acclimated to 20°C cold acclimated (CA, gray bars, *n* = 6) or 27°C warm acclimated (WA, black bars, *n* = 6) at assay temperatures of 32, 27, and 22°C. Values are means ± SE. Differing letters denote statistically significant differences between assay temperatures for each state, as determined by 2-way repeated measures ANOVA, *P* < 0.05. There were no significant differences between acclimation groups for any of the parameters.

We investigated the bioenergetics of isolated liver mitochondria from cold and warm acclimated animals that were normothermic when sacrificed. We mimicked the variation of T_b_ by changing experimental assay temperatures from 22 to 32°C (Figures 2A–D). Between acclimation groups, we found no differences in any of the respiration states. As expected, mitochondrial respiration decreased significantly with decreased assay temperature by about 40% (two-way repeated measures ANOVA, *P* < 0.01). The reduction of respiration without respiratory control (state 3 and FCCP) suggests an average Q10 of 2.84 ± 0.65 (SE) of substrate oxidation. However, respiratory control (state3/4), as a rough estimate for mitochondrial coupling and efficiency, was compromised only at 22°C (Figure 2E, two-way repeated measures ANOVA, *P* < 0.01).

### Mitochondrial proton leak kinetics

**Figure 3:**
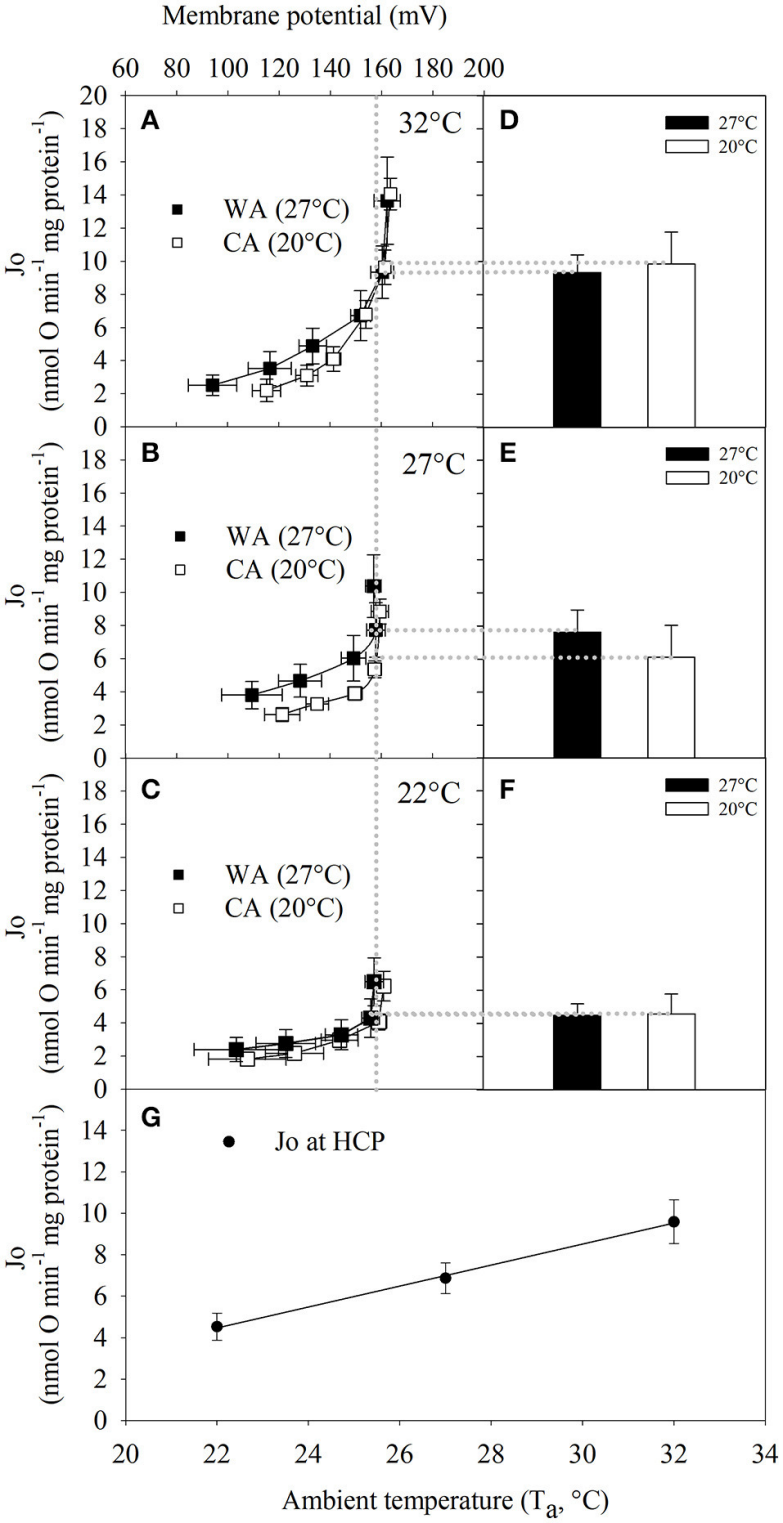
The full kinetic response of proton leak rate to changes in membrane potential of liver mitochondria from *Echinops telfairi* acclimated to 20°C (CA, white squares, *n* = 6) or 27°C (WA, black squares, *n* = 5) at assay temperatures of **(A)** 32, **(B)** 27, and **(C)** 22°C in the presence of 1 μg/ml oligomycin. **(D–F)** Mean ± SE of proton leak respiration (Jo) at the highest common membrane potential across all T_a_ (HCP = 157 mV, vertical dotted line). **(G)** Proton leak respiration of HCP at different assay temperatures (acclimation groups combined). Values are means ± SE. Least squares regression: y = −0.5059x + 20.659, where the slope of the regression was significantly different from zero (ANOVA, *P* = < 0.001).

To test whether the differences in state 4 (proton leak respiration) are caused by differences in proton conductance, or whether they are just a consequence of altered substrate oxidation (Keipert and Jastroch, 2014), we determined the proton leak kinetics of isolated tenrec liver mitochondria by simultaneous measurement of oxygen consumption and mitochondrial membrane potential. Plotting oxygen consumption driving the proton leak vs. membrane potential (Figures 3A–C), we found no statistical differences of proton leak at the highest common membrane potential between acclimation groups irrespective of assay temperature (Figures 3D–F). The proton leak at the HCP across the different T_a_, decreases significantly with decreasing assay temperature (data for acclimation groups combined, Figure 3G), demonstrating reduced proton conductance at colder temperatures by passive thermal effects.

### Mitochondrial coupling efficiency

**Figure 4:**
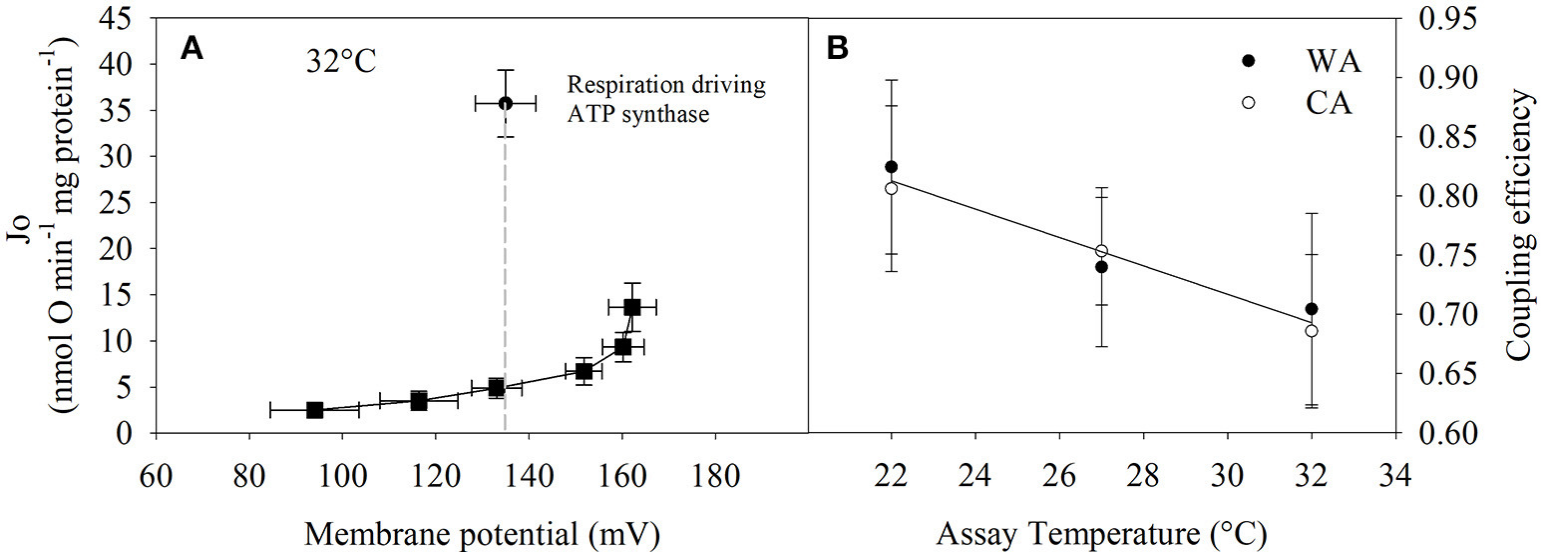
Calculation of coupling efficiency that is defined as the respiratory fraction that drives the ATP synthase. **(A)** Single example how coupling efficiency is assessed per individual. State 3 and proton leak respiration at the state 3 membrane potential (indicated by the dashed line) are required. **(B)** Coupling efficiency = (state 3—proton leak respiration)/ (state 3 respiration at state 3 membrane potential). 20°C acclimated (CA, white circles, *n* = 3) vs. 27°C acclimated (WA, black circles, *n* = 4) groups were not significantly different (two-way repeated measures ANOVA). Therefore, the linear regression analysis was performed on the combined data from both acclimation groups; y = −0.012x + 1.077 (ANOVA, *P* = 0.064). Values are means ± SE.

Next, we precisely determined coupling efficiency, which represents the proportion of oxygen consumption driving the ATP synthase of total respiration (i.e., corrected for proton leak respiration). The proportion of proton leak was determined from proton leak kinetic curves at the state 3 membrane potential (see Figure 4A). The proportion of respiration to drive ATP synthesis at 32°C is about 70% and although statistically not significantly different, trends to increase up to 85% at lower temperatures (Figure 4B).

## Discussion

In this study, we report on adjustments of mitochondrial bioenergetics allowing energy homeostasis in the lesser hedgehog tenrec, a protoendothermic eutherian, during its T_b_ cycles. We find that the mitochondrial system maintains mitochondrial efficiency at lower assay temperatures, partially by decreases in proton leak, counteracting decreases in temperature-dependent substrate oxidation rates.

While these passive thermal effects appear to control mitochondrial energy turnover, cold acclimation of the tenrec had neither effect on the *in vivo* physiology such as body mass, T_b_ and metabolic rate, nor on bioenergetics parameters of isolated liver mitochondria.

The patterns of T_b_ to acute changes in T_a_ (Figure 1) as described previously (Oelkrug et al., 2013) clearly support the observations of a distinct ectotherm-like nature where T_b_ closely matches changes in T_a_ (Scholl, 1974; Lovegrove and Génin, 2008). This was particularly true for cases where animals entered torpid-like minimal metabolic states during the experiments (Figure 1C). Here, the correlation between T_b_ and T_a_ was very close to 1. Although the familiar Scholander-Irving model of thermoregulation is typically only applied to euthermic endotherms (McNab, 2002), metabolic heat production was adjusted similarly to euthermic endotherms below the TNZ when measuring RMR in the tenrecs (Figure 1B), clearly demonstrating the ability of classical euthermic, eutherian thermoregulation. Nevertheless, T_b_ in the tenrecs is amongst the lowest within mammals and similar to monotremes (Nicol, 2017). Interestingly, when undisturbed, tenrecs will drop their metabolic rates to a minimum, while adjusting their T_b_ to T_a_. Even though the reduction in MR_min_ with changes in T_a_ that we observed here (Figure 1B) was only marginal in comparison to changes in RMR, the continuous reduction in MR_min_ appears relevant given the continuous reduction in T_b_. It remains to be investigated whether passive heat loss is the major driver in the reduction of T_b_ as is the case in many species entering torpor (Nicol and Andersen, 2007; Oelkrug et al., 2011), rather than the controlled reduction in MR and hence T_b_.

While our understanding of the overarching thermoregulatory patterns of tenrecs has become clearer, the cellular basis supporting this unique pattern is not well-understood. The bioenergetics of liver mitochondria are well-established nominators of (basal) metabolic rates (Porter and Brand, 1995; Polymeropoulos et al., 2012) and actively adjust in response to hypothermic states in some rodent species (Staples and Brown, 2008; Kutschke et al., 2013). Thus, investigating temperature-dependent bioenergetics in the frequently heterothermic tenrec may yield relevant insights into concepts and mechanisms that occurred during early evolution of eutherian metabolism. Therefore, we investigated the flexibility of mitochondrial function in response to acute and chronic temperature changes in isolated liver mitochondria of tenrecs. In contrast to some ectothermic vertebrates such as fish and amphibians, where mitochondrial state 4 respiration and proton leak rate is suppressed in chronic cold (Jastroch et al., 2007; Trzcionka et al., 2008), acclimation temperature had no such impact on liver mitochondrial proton leak in the tenrec (Figure 3). This finding may indicate that there are no functional, long-term changes accompanied by cold acclimatization. In our experiments, we sacrificed the animals at normothermic temperatures for experimental consistency. Under these experimental conditions, putative, rapid molecular changes in response to hypothermic or hypometabolic states in torpor may be missed.

The capacity of ATP production is cold-sensitive with a 50% reduction of respiration rates in state 4 (Figure 2C) and 70% reduction in state 3 over a 10°C temperature decrease (32 vs. 22°C, Figure 3B). Similar findings, but with lesser physiological significance, have been reported for rat liver mitochondria, where a 12°C decrease in assay temperature resulted in a 50% decrease of state 3 and 4 respiration rates (Dufour et al., 1996).

For tenrec mitochondria, the respiratory control ratio at 22°C was 40% lower than that at 27 and 32°C (Figure 2E). However, the correction of ATP-linked state 3 for proton leak respiration at the state 3 mitochondrial membrane potential reveals maintenance of mitochondrial efficiency of about 70–85%, strongly trending to increase at lower temperatures (Figure 4B). This is a striking finding demonstrating an inherent thermal plasticity of tenrec mitochondria that favors a thermoregulatory strategy allowing optimal mitochondrial function in heterothermic states that are naturally experienced by tenrecs. If this may be a trait unique to tenrecs or protoendotherms remains to be elucidated. Clearly, more data are required to phylogenetically generalize this suggestion.

Within vertebrates, the level of the basal mitochondrial proton leak of *E. telfairi* is at an intermediate level between endo- and ectotherms (Polymeropoulos et al., 2017). However, on a phylogenetically informed basis, the tenrec mitochondrial proton leak scales within other eutherian mammals but not reptiles, corroborating that the molecular, mitochondrial setup is “mammalian” while the behavioral pattern could be classified as “reptilian.” It would therefore be interesting to study whether mitochondrial efficiency at colder temperatures in ectothermic vertebrate species exhibits a similar pattern, thus being a plesiomorphic trait, or whether these mechanistic adjustments were derived in the need to adjust to endothermic metabolism.

In conclusion, the bioenergetic setup of tenrec liver mitochondria resembles mammalian and ectothermic features. Mammalian features are reflected in the amplitude of tenrec liver proton leak within the eutherian range, while increased mitochondrial efficiency may have enabled to cope with energy conversion at low T_b_. The latter possibly enables the unique thermoregulation of the tenrec with exceptionally long ectothermic episodes—an assumed prerogative of eutherian survival at the Cretaceous-Paleogene boundary.

## Author contributions

EP and RO equally contributed to this manuscript. RO and MJ conceived the ideas, RO conducted the experiments, EP, RO, and MJ prepared the manuscript and figures and analyzed the data, and approved the manuscript.

### Conflict of interest statement

The authors declare that the research was conducted in the absence of any commercial or financial relationships that could be construed as a potential conflict of interest.
